# Welcome Message from the Editor-in-Chief

**DOI:** 10.3390/jof1010001

**Published:** 2014-07-16

**Authors:** David S. Perlin

**Affiliations:** Public Health Research Institute (PHRI), International Center for Public Health, New Jersey Medical School, Rutgers, the State University of New Jersey, 225 Warren Street, Newark, NJ 07103, USA; E-Mail: perlinds@njms.rutgers.edu; Tel.: +1-973-854-3200; Fax: +1-973-854-3101

Fungi are one of the most important and diverse groups of organisms on the planet, having a dual impact on humanity. They adversely impact human and animal health and can be a scourge to agriculture, while in turn serving as a beneficial source for foods and beverages, new medications, and biocontrol. There are approximately 1.5 million different species of fungi on Earth, which largely reside in soil and plant. They are also readily found on human skin and within the gastrointestinal and genitourinary tract, yet only about 300 species are known to make people sick [1,2]. Fungi are bountiful in the environment and we encounter them everyday, usually in the form of freely dispersed spores and hyphal fragments that we breath-in. Typically, encounters with fungi are harmless, as the human immune systems is well poised to handle such interactions. However, some fungal species pose significant health risks, such as endemic mycoses or those producing toxins like mycotoxins. Most importantly, immune dysfunction can lead to serious life-threatening diseases or severe fungal-induced allergic diseases such as asthma or other chronic conditions [3]. In fact, most invasive fungal diseases are associated with changes in the host such as immunosuppression, antibiotic-mediated disruption of microflora, or other immunosuppressing conditions resulting from HIV/AIDS and hematologic malignancies [3,4]. Such diseases require therapy with antifungal agents. Yet, there are only limited classes available to treat invasive fungal infection, and emerging drug resistance further restricts treatment options. In some cases, agents used to control agriculturally important moulds are the same class as those used to treat humans, and *de novo* resistance can emerge from the environment [5]. Fungi are not always easy to detect and cryptic chronic infections in the form of unculturable organisms can confound diagnosis [6].

Fungi also contribute significantly to managing human and animal diseases by producing major antiinfectives, immunosuppressant drugs, and anticholesterol statins. As they synthesize complex molecules using well-defined metabolic pathways, fungi are valuable for biotechnology. Their genetic manipulation and modulation of environmental growth conditions influence product formation. The production of soy, cheese, bread, wine, and beer have greatly benefited from modification of such factors, as they improve yield and food quality. Large-scale production and industrial application of fungal proteins makes higher fungi a valuable source of commercially significant proteins.

There are many challenges to understand fungi in their natural environment, and the biological changes that occur when fungi adapt to new environments, especially within the human and animal hosts and in response to antifungal agents. We need to improve our methods to detect them in acute and chronic diseases, and we need to control and influence their growth in medical and agricultural settings. In this context, it is with great pleasure that we celebrate the launch of the *Journal of Fungi* (*JoF*) with this inaugural issue. On behalf of the editorial team, I would like to extend a very warm welcome to the readership of *JoF*. I want to take this opportunity to thank all of our authors, editors and dedicated reviewers, who will contribute to the success of the journal.

The *JoF* is an international, peer-reviewed scientific open access journal that provides an advanced forum for studies related to pathogenic fungi, fungal biology, and all other aspects of fungal research. It is our objective to advance fundamental knowledge of fungal biology, genetics and physiology, and address the growing importance of fungi in medicine, public health, health policy and security, agriculture, and the environment through innovative science and leading edge opinions.

The journal publishes reviews, regular research papers, and communications in quarterly issues. It is our aim to encourage scientists to publish their methodological details, experimental and theoretical results in as much detail as possible. Therefore, there is no restriction on paper length and associated supplemental materials.

In addition to conventional contributions from the academic community, we encourage contributions from industry and public health professionals. Guest editors with novel theme concepts are always welcome and, of course, we encourage comments and suggestions that could improve the quality of the journal.

We hope you will find the *JoF* a valuable and influential voice to advance our understanding fungal sciences and the global importance of fungi.

Thank you!


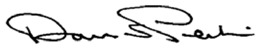

David S. Perlin

